# Coexistence of acute pancreatitis and an obstructing upper ureteral calculus: does a calculus cause pancreatitis? a case report

**DOI:** 10.1097/MS9.0000000000000897

**Published:** 2023-05-23

**Authors:** Rawa Bapir, Karokh F. Hama Hussein, Hiwa O. Baba, Aso S. Muhialdeen, Soran H. Tahir, Berun A. Abdalla, Shvan H. Mohammed, Abdulwahid M. Salih, Fahmi H. kakamad, Ismaeel Aghaways

**Affiliations:** a Smart Health Tower, Madam Mitterrand Street; b Department of Urology, Surgical Teaching Hospital; c Kurdistan Center for Gastroenterology and Hepatology; d Kscien Organization, Hamdi Str, Azadi Mall; e College of Medicine, University of Sulaimani, Madam Mitterrand Street, Sulaimani, Kurdistan, Iraq

**Keywords:** case report, hydronephrosis, pancreatitis, renal impairment, ureteric stone

## Abstract

Acute pancreatitis is an inflammatory disease that can affect both the peripancreatic tissues and distant organs. There are few reports of acute pancreatitis caused by endourological procedures. However, an obstructing ureteral calculus causing acute pancreatitis is very rare.

**Case presentation:**

A 36-year-old male patient presented with epigastric, and right loin pain with decreased urine output for 3 days. On physical examination, he had central abdominal and right flank tenderness. An abdominal ultrasound showed mild to moderate ascites, a hyperechoic pancreas, a small (26×77 mm) left kidney with increased echogenicity, right renal hypertrophy with moderate hydronephrosis, and a dilated upper ureter due to a 10 mm obstructing stone with a perirenal fluid collection. The diagnosis of acute pancreatitis with an obstructing right upper ureteric stone was established. Under spinal anesthesia, an emergency ureteroscopy with laser fragmentation of the stone was performed, and a JJ stent was inserted. He developed postobstructive diuresis and his renal function was improved with a rapid decline of pancreatic enzymes as well.

**Clinical discussion:**

Two theories explain the presentation of acute pancreatitis by ureteral obstruction. First, the obstructed severe hydronephrotic kidney compresses the duodenum and head of the pancreas, obstructing the distal part of the common bile duct and triggering the elevation of pancreatic duct pressure, bile reflux, trypsin activation, and pancreatic autodigestion. The second theory states that acute pancreatitis develops when urine is extravasated from an obstructed kidney into the adjacent tissues, irritating the uncinate process of the pancreas.

**Conclusion:**

Although mentioning ureteral obstruction as a cause of pancreatitis is scarce, the clinician should be aware that in each case of ureteral obstruction, the emergence of acute pancreatitis is a possible complication.

## Introduction

Highlights

- Acute pancreatitis is an inflammatory disease affecting both the peripancreatic tissues and distant organs.
- The mortality rate is about 10–30% in severe cases.
- Obstructing ureteral calculus causing acute pancreatitis is very rare.
- To the best of our knowledge, only three reports are available in the literature.

Acute pancreatitis is an inflammatory disease that can affect both the peripancreatic tissues and distant organs, with a mortality rate of about 10–30% in severe cases^1,2^. It comprises about 5 to 73 cases per 100 000 persons across the world^3^. The disease is classified into two types: interstitial edematous pancreatitis and necrotizing pancreatitis. The vast majority of patients with acute pancreatitis experience pancreatic enlargement due to inflammatory edema that can be either diffuse or localized. Necrotizing pancreatitis is typically identified by necrosis that can affect both the pancreas and peripancreatic tissues^4^. Acute pancreatitis is a multifaceted process that originates in the pancreatic acinar cells. The precise mechanisms that trigger its development are still under debate; however, the preferred hypothesis is the injury or disruption of the pancreatic acini, which allows pancreatic enzymes to leak into the surrounding pancreatic tissue^5^. Alcohol consumption or biliary diseases are among the common causes in most cases. Unusual causes like drugs, trauma, and viral infections can be present in up to 20% of the cases^2^. Although alcohol consumption is not the only cause of pancreatitis, it does seem to increase the pancreas’ vulnerability to injury from other causes, like genetic or environmental factors, thereby increasing the risk of developing acute pancreatitis^6^. In urology, there are few reports of acute pancreatitis caused by extracorporeal shock-wave lithotripsy for ureteral or renal stones, ureteropelvic junction obstruction, postureteroscopy for ureteral stones, and severe hydronephrosis causing obliteration of the distal common bile duct^7–10^. However, an obstructing ureteral calculus causing acute pancreatitis is very rare, and to the best of our knowledge, only a few reports are available in the literature^2,11^. This study aims to report a case of simultaneous right-sided ureteral obstruction caused by a stone and acute pancreatitis in a young man with a functionally solitary right kidney. This report has been written according to the Surgical CAse REport (SCARE) guideline^12^.

## Case presentation

### Patient information

A 36-year-old male patient presented to the gastroenterology clinic with epigastric and right loin pain, along with decreased urine output, for 3 days. He also reported associated symptoms of nausea, vomiting, and loss of appetite. There was no history of alcohol consumption or chronic medical illnesses. Examination of the vital signs revealed tachycardia (110 bpm), tachypnea (20 cycles/minute), and a blood pressure of 100/60 mmHg. On physical examination, he had central abdominal and right flank tenderness.

### Clinical findings

Blood investigations were as follows: white blood cells (27.4×109/l), hemoglobin 12.2 gm/l, C-reactive protein 44.3 mg/l, creatinine (22.8 mg/dl), urea (196 mg/dl), amylase (1103 IU/l), lipase (1603 IU/l), alkaline phosphatase 115 IU/l, alanine aminotransferase 38.5 IU/l), random glucose (122 mg/dl), Na+ (140 mmol/l), K+ (5 mmol/l), Cl- (106 mmol/l) and triglycerides (141 mg/dl), calcium (9 mg/dl), parathyroid hormone 55 IU/l, and urinalysis showed microscopic hematuria (25 RBC/HPF) without pyuria and cast cells.

### Diagnostic assessment

An abdominal ultrasound (U/S) showed mild to moderate ascites, a hyperechoic pancreas, a small (26×77 mm) left kidney with increased echogenicity, right renal hypertrophy with moderate hydronephrosis, and a dilated upper ureter due to a 10 mm obstructing stone with a perirenal fluid collection. While the liver, gall bladder, and common bile duct were normal. The diagnosis of acute pancreatitis with an obstructing right upper ureteric stone was established. Once the gastroenterologist could not find a cause for pancreatitis other than the obstructing stone, he referred him to the urology department.

### Therapeutic intervention

Because computed tomography (CT) kidneys, ureters and bladder was unavailable at the time of presentation, and the patient’s condition was deteriorating, consent was obtained from the patient to perform either an emergency right percutaneous nephrostomy or a right JJ stent insertion, as well as hemodialysis as needed. Owing to the risk of bleeding, a trial of double J stent insertion was preferred. Under spinal anesthesia, the patient underwent a trial of right JJ stent insertion. However, the guide wire could not pass to the kidney due to stone impaction. A ureteroscopy with laser fragmentation was done to aid JJ stent insertion, and a few stone fragments were pushed back to the kidney, then the right double J stent was inserted.

Following the operation, the patient developed immediate postobstructive diuresis. In the first 24 h, he passed around 24 l of diluted urine, and serum creatinine decreased to 11 mg/dl. The patient received 1–2 l of IV fluids at a rate of 250–500 ml/hour. Oral feeding was resumed cautiously with low-protein meals due to the patient’s elevated creatinine levels. In addition, the patient was administered meropenem (500 mg twice daily), analgesics, and antipyretics (paracetamol 1 g, three times a day).

### Follow-up

**Figure 1:**
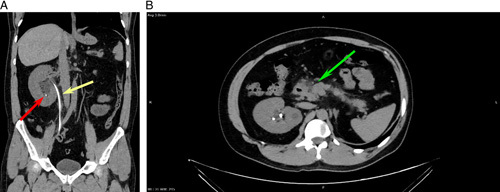
A, Coronal section CT KUB, shows JJ stent in right ureter ‘yellow arrow’, still there is mild pelvicalyceal dilation, small stone in lower pole calyx ‘red arrow’. B, Axial section of CT KUB, shows edema and fat stranding between the head of the pancreas and the second part of the duodenum, with a feature of acute pancreatitis.

He was kept on close follow-up with monitoring fluid balance, urine output, and careful monitoring of serum electrolytes. He responded well to the treatment, and on the fifth postoperative day, his blood investigations showed: serum creatinine 1.1 mg/dl, blood urea 25 mg/dl, serum amylase 379 IU/l, and serum lipase 834 IU/l. On the sixth postoperative day, he was discharged home in good general condition. A CT KUB on the 14th postoperative day showed an edematous pancreas, a double stent well-positioned, and a 4 mm lower calyceal stone (Fig. 1). However, on presentation, the case denied a history of diabetes, and his blood glucose was within the normal range, he was subsequently diagnosed with diabetes mellitus and put on metformin 500 mg. Six weeks later, the double J stent was removed, and the patient was kept on regular follow-ups for the management of diabetes mellitus. During the patient’s 3-month follow-up after management, his lab results showed normal levels of C-reactive protein (2.86 mg/l), urea (36.3 mg/dl), amylase (81 IU/l), and lipase (42.3 IU/l). The creatinine level decreased significantly from 11 mg/dl to 1.37 mg/dl, and his blood glucose levels were well-controlled with a dose of 500 mg metformin taken twice daily. After 2 years of routine follow-up, the patient had fully recovered.

## Discussion

The major risk factors for acute pancreatitis include bile/pancreatic duct obstruction, tumors, drugs, lipid abnormalities, and iatrogenic events (as a postoperative complication)^13^. Alcohol consumption increases the risk of pancreatitis by multiple folds^14,15^. Regarding the few reports available in the literature, the common clinical presentations of acute pancreatitis induced by a ureteric obstruction are acute abdominal pain radiating to the back, abdominal tenderness, nausea, vomiting, and pain in the flank, loin, and epigastric region^2,10,11^. In the present case report, the patient complained of epigastric and right loin pain for several days, associated with nausea, vomiting, loss of appetite, and decreased urine output without having a fever. No risk factors were identified, and even the history of alcohol consumption was negative. To the best of our knowledge, our case is the fourth reported case in the literature. A summary of several previous studies on pancreatitis has been demonstrated in Table 1. In 2009, Raj and Murray^2^ reported a 77-year-old man with acute pancreatitis due to an obstructing right-side ureteropelvic junction stone that responded to the immediate decompression of the obstructed kidney by a percutaneous nephrostomy. Later on, Dospinescu *et al.* ^11^ reported a 55-year-old female patient with acute focal pancreatitis due to an obstructing left upper ureteric stone with extensive periureteral and perinephric stranding and infiltration on CT KUB. Similarly, the current case had an obstructing right upper ureteric stone causing a back pressure effect with the presence of perirenal and abdominal free fluid collection on the U/Sscan. However, unlike in previous cases, he had very high blood urea and serum creatinine on presentation (blood urea 196 mg/dl and serum creatinine 22.8 mg/dl), and the contralateral kidney was small with increased echogenicity. The presence of a functionally solitary kidney in our patient may have precipitated the onset of pancreatitis. However, a DTPA renal scan was not done to exactly determine the differential renal function, which is a limitation of our study.

**Table 1:** Summary of several studies of pancreatitis

References	Sex	Age (Years)	Chief complaint	Diagnosis	Management
Raj *et al.* ^2^	Male	77	Acute abdominal pain, nausea, and vomiting.	Elevated lipase, amylase, and impaired renal function. A CT scan confirmed right-sided ureteric obstruction.	Percutaneous nephrostomy and shock-wave lithotripsy
Twohig *et al.* ^3^	Male	60	Periumbilical pain and hematuria	Tachycardia, tachypnea, abnormal complete blood count, and elevated lipase. A CT scan confirmed severe necrotizing pancreatitis.	Left ureteral stent placement and Percutaneous nephrostomy.
Feng *et al.* ^5^	Female	77	Abdominal pain, nausea, and vomiting	Elevated amylase and bacterial infection. A CT scan confirmed acute pancreatitis and a diethylenetriamine penta acetic acid renal scan with furosemide and retrograde pyelogram confirmed obstruction of the left ureteropelvic junction.	Intravenous antibiotics and Percutaneous nephrostomy.
Park *et al.* ^6^	Male	49	Fever and decreasing mental activity	Elevated lipase, amylase, and bacterial infection. A CT scan showed perinephric inflammatory fluid collection and extension to the tail of the pancreas.	Antibiotics
Dospinescu *et al.* ^7^	Female	55	Left loin pain and nausea	Abnormal blood test results. Clinical and radiological diagnoses confirmed acute focal pancreatitis caused by an obstructing ureteric stone.	Antibiotics, fluids, analgesia, ureteroscopy, and ureteral stent placement.
Morehouse *et al.* ^9^	Male	63	Abdominal pain, nausea, and vomiting	A CT scan demonstrated pancreatic inflammation encasing the ureter and a post-CT radiograph confirmed hydronephrosis.	Unmentioned
Morehouse *et al.* ^9^	Male	42	Abdominal pain, nausea, and vomiting	A CT scan demonstrated pancreatic inflammation partially obstructing the right ureter.	Unmentioned
Morehouse *et al.* ^9^	Male	43	Abdominal pain	Elevated amylase. A CT scan revealed extensive pancreatic calcification and extensive pancreatic phlegmon.	Surgical drain
Morehouse *et al.* ^9^	Male	34	Abdominal pain	CT scan showed pancreatitis and right-sided ureteral obstruction.	Unmentioned
Morehouse *et al.* ^9^	Female	66	Pancreatitis	Elevated amylase. A CT scan showed pancreas head enlargement and the right renal collecting system mild dilatation.	Exploratory laparotomy
Morehouse *et al.* ^9^	Female	58	Acute pancreatitis	A CT scan showed pancreas enlargement, bile duct, and right renal pelvis dilatation.	Unmentioned
Lugito *et al.* ^11^	Male	38	Right-sided flank and epigastric pain, nausea, and vomiting	Urinary tract infection, elevated lipase, and amylase. Both ultrasound and MRCP confirmed hydronephrosis with right-sided multiple nephrolithiasis.	Percutaneous nephrostomy

Two theories have explained the occurrence of acute pancreatitis caused by ureteral obstruction. First, the obstructed severe hydronephrotic kidney compresses the duodenum and head of the pancreas, obstructing the distal part of the common bile duct and triggering the elevation of pancreatic duct pressure, bile reflux, trypsin activation, and pancreatic auto-digestion. The second theory states that acute pancreatitis develops when urine is extravasated from an obstructed kidney into the adjacent tissues, irritating the uncinate process of the pancreas^10^. The current case supports the latter theory as the U/S scan showed moderate hydronephrosis and perinephric fluid collection.

The key features of acute pancreatitis are abdominal pain and a threefold elevation of amylase and lipase levels above the normal range^10^. Our case had more than threefold increases in both enzymes (serum amylase 1103 IU/l, normal range 28–100, and serum lipase 1603 IU/l, normal range (13–85), confirming the diagnosis of acute pancreatitis. According to Raj and Murray^2^, abdominal pain can be a confounding factor between renal tract infection and pancreatitis, potentially leading to a delayed diagnosis of the latter disease. On the other hand, Lugito *et al.* ^10^reported that normalizing pancreatic enzymes following obstructed urinary system decompression through percutaneous nephrostomy confirms that ureteric obstruction is a factor in the development of acute pancreatitis.

Several diagnostic modalities exist for acute pancreatitis. The U/S can detect gallstones, dilatation of the bile duct, and free peritoneal fluid. However, it is not sufficiently sensitive for determining the grade of pancreatitis or detecting the presence of necrosis. On the other hand, contrast-enhanced CT scan is vital for detecting acute pancreatitis and ureteral obstruction. Magnetic resonance cholangiopancreatography is a noninvasive diagnostic technique for imaging the pancreaticobiliary tree without the need for contrast media^16,17^. In terms of management, Raj and Murray^2^ used an 8.5-Fr percutaneous nephrostomy to decompress the obstructed urinary system and treat sepsis. Following this, they performed shock-wave lithotripsy three weeks later to manage the obstructing ureteric calculus. Another study also utilized percutaneous nephrostomy for management, but the patient passed away due to disseminated intravascular coagulation and sepsis^10^. Furthermore, Dospinescu *et al.* initially treated their case with antibiotics, fluids, and analgesia. Subsequently, in response to the patient’s complaint of renal colic, they performed an emergency ureteroscopy to extract the stone and inserted a ureteric stent for a period of 4 weeks^11^. In the current report, U/S revealed mild to moderate ascites, a hyperechoic pancreas, a small left kidney with increased echogenicity, and right renal hypertrophy with moderate hydronephrosis and a dilated upper ureter due to a 10 mm obstructing stone. Nevertheless, we could not perform a CT scan on the presentation due to its unavailability, and the patient had renal impairment. We immediately proceeded to an emergency JJ stent insertion. Two weeks after the operation, a CT KUB showed an edematous pancreas.

## Conclusions

Although mentioning ureteral obstruction as a cause of pancreatitis is scarce, the clinician should be aware that in each case of ureteral obstruction, the emergence of acute pancreatitis is a possible complication.

## Ethical approval

None.

Consent to participate: Written informed consent was obtained from all participants or the parents in the case that the participants were underage.

## Consent

Written informed consent was obtained from the patient for the publication of this case report and accompanying images. A copy of the written consent is available for review by the Editor-in-Chief of this journal on request.

Patient consent for publication: Not applicable.

## Sources of funding

No funding was received.

## Author contribution

R.B. was the urologist who managed the case and was one of the major contributors to the study. K.F.H.H., H.O.B., A.S.M. conducted the literature review. S.H.T. conducted the radiological examination and interpretations. A.M.S. and F.H.K. were involved in the manuscript drafting. B.A.A., S.H.M., and I.A. were involved in the critical review, and improvement of the manuscript. All of the authors were involved in the final approval of the study.

## Conflicts of interest

The authors declare that they have no competing interests.

## Research registration (for case reports detailing a new surgical technique or new equipment/technology)

None.

## Guarantor

Fahmi H. Kakamad.

## Provenance and peer review

Not commissioned, externally peer-reviewed.

## Data availability statement

Not applicable.

## Patient perspective

The patient was completely satisfied with the management approach and did not have any complaints about it. Additionally, the patient praised the staff for their efforts during his treatment.
